# Multiple, Large Colonic Xanthomas Presenting as Intestinal Obstruction

**DOI:** 10.7759/cureus.8953

**Published:** 2020-07-01

**Authors:** Zarak H Khan, Kashif Mukhtar, Syeda Ramsha Zaidi, Munis M Ahmed

**Affiliations:** 1 Internal Medicine, St. Mary Mercy Hospital, Livonia, USA; 2 Internal Medicine, King Edward Medical University, Lahore, PAK

**Keywords:** colonic xanthomas, colonic mass, intestinal obstruction

## Abstract

Gastrointestinal (GI) xanthomas are rare entities found incidentally on endoscopy. There have been only a few cases where they presented with symptoms of bowel obstruction. We present a case of an 89-year-old woman with a history of short-gut syndrome due to partial colectomy who had multiple admissions with recurrent nausea/vomiting, abdominal distension, and bloating. She was found to have multiple, large, mass-like xanthomas in the rectosigmoid colon. The unusual location, mass-like lesions, and large size led to luminal narrowing causing the patient to have obstructive symptoms, which is a very unusual presentation of colonic xanthomas as most are discovered incidentally.

## Introduction

Xanthomas are yellowish-white lesions found in different parts of the body especially on the skin. These lesions are due to the accumulation of fat tissue. Under the microscope, xanthomas consist of foamy histiocytes [1]. The most common site of xanthomas in the gastrointestinal (GI) tract is the stomach, predominantly in the antrum and the pyloric region [2]. The esophagus and small intestines are usually spared [3]. The rarest of all are colonic xanthomas with only a few cases found in the literature [4]. In many cases, there are no underlying conditions that predispose patients to xanthomas like familial or acquired disorders of fat metabolism or lymphoproliferative disorders [5]. This case has previously been presented at the World Congress of Gastroenterology organized by the American College of Gastroenterology (ACG) in October 2019 [6].

## Case presentation

**Figure 1:**
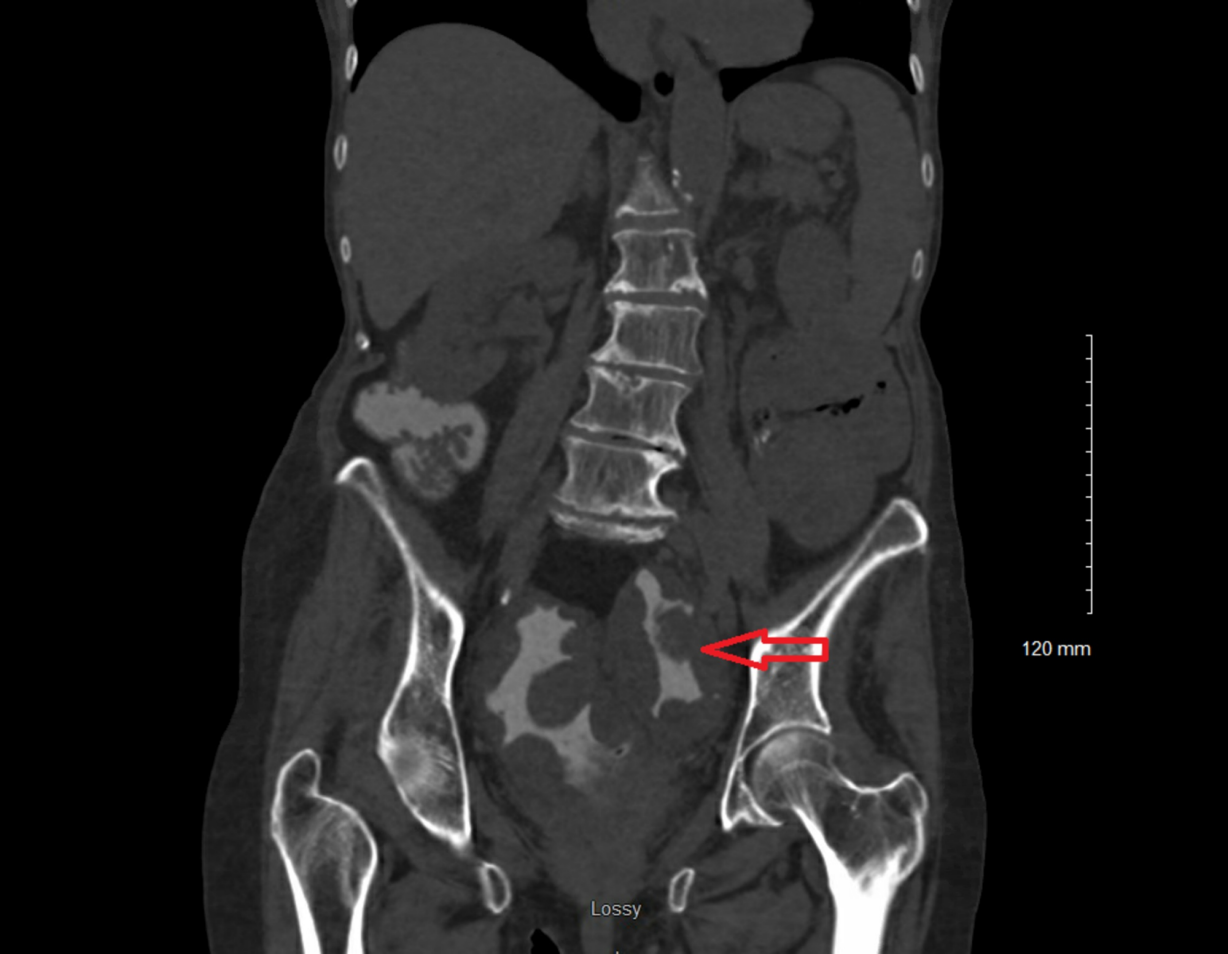
CT scan of the abdomen, arrow reveals severe thickening of the colon (red arrows).

**Figure 2:**
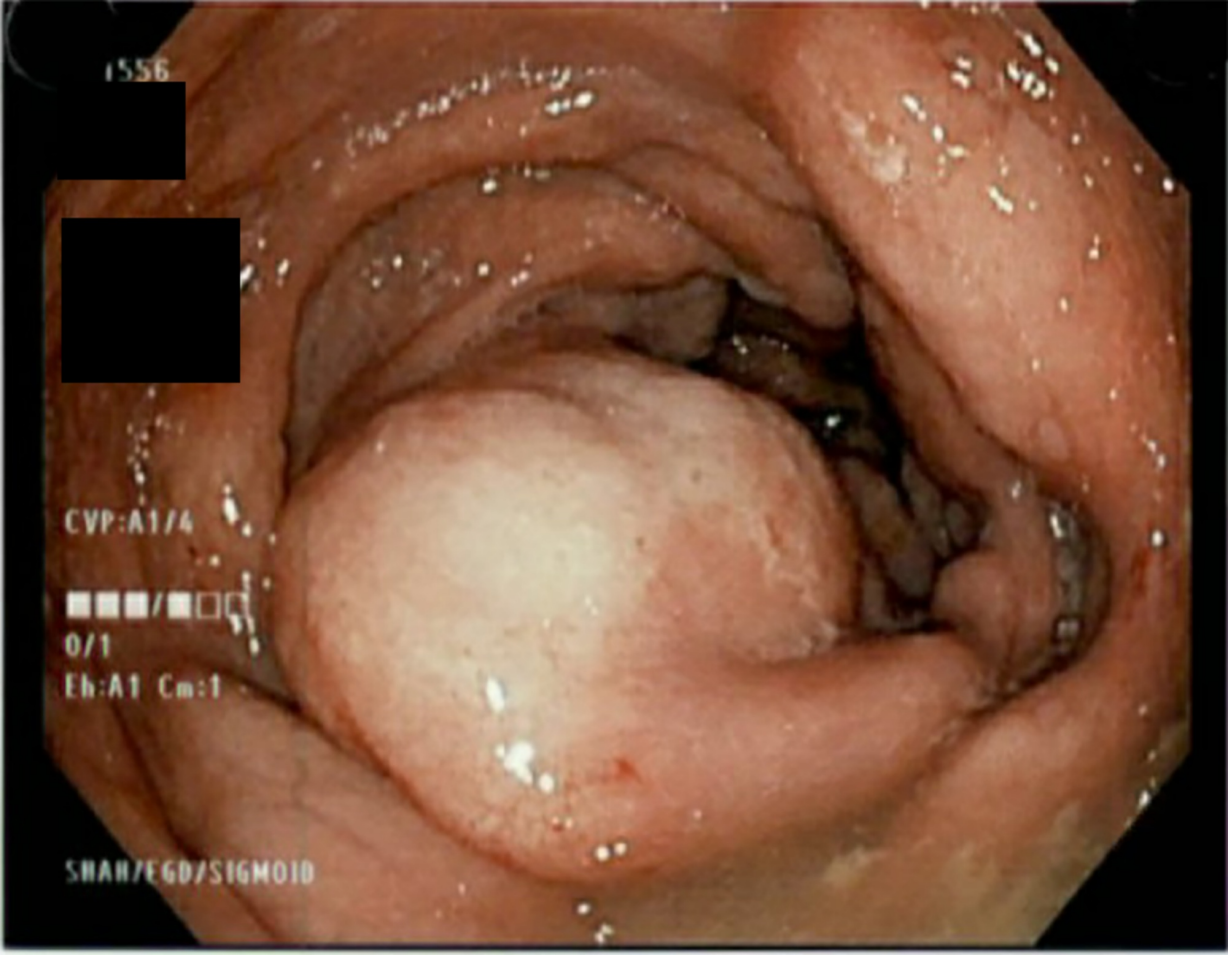
Sigmoidoscopy showing submucosal mass-like lesion.

**Figure 3:**
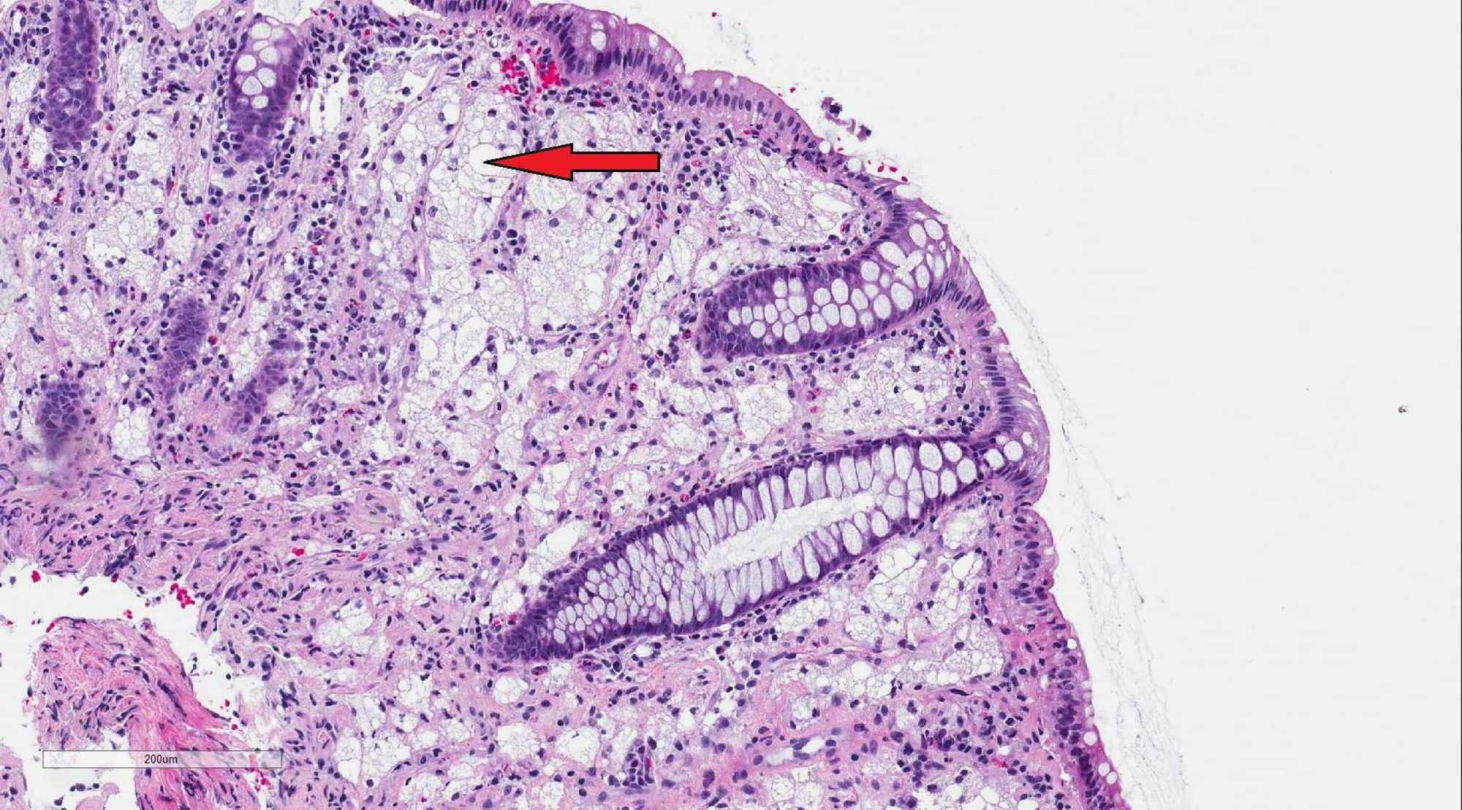
Histopathological exam of the mass-like lesion showing foamy histiocytes with neutral fat in the lamina propria (red arrow), finding consistent with xanthoma.

This is a case of an 89-year-old woman, with a past medical history of short-gut syndrome secondary to partial colectomy at age 58, who presented to the hospital with complaints of nausea and vomiting of three days duration accompanied with generalized weakness. Upon presentation, her vitals were normal. On examination, her abdomen appeared distended with hyperactive bowel sounds. Blood work was notable for hyponatremia with sodium of 120 mEq/L. A CT scan of the abdomen revealed marked wall thickening of the rectosigmoid colon consistent with diffuse colitis (Figure 1). She underwent an endoscopy which was unremarkable. Due to the strong suspicion for obstruction and underlying findings on CT imaging, she underwent sigmoidoscopy which revealed multiple submucosal masses in the rectum and distal sigmoid, highly suspicious for malignancy (Figure 2). Biopsy specimens taken from the masses revealed xanthomatous mucosal changes without any evidence of malignancy or inflammation (Figure 3).

A fasting lipid panel was obtained which was within normal limits. The patient's vomiting and electrolyte abnormalities resolved with bowel rest and IV fluids. Upon chart review, the patient had previously been admitted to the ED with similar complaints on multiple occasions. During each visit, imaging was significant for colitis, and colonoscopy had revealed sigmoid masses proven to be xanthomas on histopathology. The patient was given bowel rest which resolved her symptoms and she did not have to undergo surgical intervention. On discharge, she was advised to regularly follow up with a gastroenterologist and she was given a high fiber diet by a nutritionist to help prevent recurrence of her symptoms.

## Discussion

Xanthomas have been seen in different parts of the GI tract. Although true incidence is unknown, these lesions occur more frequently in the stomach with relative sparing of other parts of the gut [7]. The large bowel is mostly spared by these lesions. They are most commonly identified incidentally on colonoscopy and usually present as nodules, plaques, or patches [8]. It is rare to see these lesions presenting as masses suspicious for malignancy [9]. Diseases other than xanthomas that present with yellow to white lesions in the colon include pseudomembranous colitis, lipomas, and lymphomas.

A major study that looked at rectosigmoid xanthomas was reported by Miliauskas that described 13 cases [10]. The study found that the incidence was greater in female patients and the mean age was 54 years. Colonoscopically, most xanthomas (70%) had a yellow hue. All cases were located in either sigmoid colon or rectum and most of the lesions had a sessile appearance.

The largest case series about colonic xanthomas has been described by Nakasono et al. who has described 28 biopsy-proven colorectal xanthomas in 25 patients [11]. In their study, the most common location of the xanthomas was in the sigmoid colon (60.7%). Most of the xanthomas had sessile appearance (82.1%). The xanthomas were microscopically found to have foam cells in the lamina propria, which helped in the identification. Histopathologically, the surface epithelium revealed hyperplastic changes in 78.6% of the cases, and the colonic glands were enlarged in 14.3% cases. The study also suggested previous mucosal injury or hyperplastic changes as likely triggers for pathogenesis of these xanthomas. Recently, Iwamuro et al. have described two cases of yellow to whitish lesions in the rectum which were identified as xanthomas [8].

The patient in our study is unique with respect to the clinical presentation and location of the lesions. While most of the above cases report incidentally found lesions, our patient presented with symptoms consistent with bowel obstruction such as nausea, vomiting, bloating, and abdominal distension. The sigmoidoscopy revealed that the xanthomas were much larger compared to previous studies, looked like masses, and might have had a mass effect (Figure 1). The CT scan showed that there might be similar lesions present near the ileocolic junction, however, these lesions were not seen due to the limitation of the sigmoidoscopy examination. Only one study to date, by Goodman has found xanthomas at the ileocecal junction which resulted in dysfunctional motility and luminal stenosis [12]. The patient had a similar presentation presenting with signs and symptoms of intestinal obstruction. 

As discussed above, there have been rare reports of colorectal xanthomas in the past but the majority of those were incidental findings in routine colonoscopy without any symptoms. Furthermore, none of the xanthomas appeared as masses. Gastroenterologists should always keep this diagnosis as a differential in mind when they encounter lesions concerning for malignancy in a patient presenting with symptoms of bowel obstruction.

## Conclusions

The GI tract xanthomas are mostly found in the upper tract. Xanthomas of the rectosigmoid colon are rare. As discussed in the case above, a patient can present with symptoms of intestinal obstruction if the xanthomas are large in size or if they are present at ileocecal junction. Xanthomas should always be in the differential diagnosis of a mass-like lesion found on endoscopy or colonoscopy. A pathological evaluation of the mass-like lesion is recommended.
